# Calprotectin (S100A8/A9) in Familial Mediterranean Fever

**DOI:** 10.1186/1546-0096-13-S1-P120

**Published:** 2015-09-28

**Authors:** R Pepper, M Hutchinson, S Henderson, D Rowczenio, P Hawkins, H Lachmann

**Affiliations:** 1 UCL, National Amyloidosis Centre, London, UK; 2 UCL, London, UK

## Introduction

Calprotectin (S100A8/A9) is an example of a damage associated molecular pattern and a TLR4 ligand. It is expressed in neutrophils, monocytes and early infiltrating macrophages. Calprotectin, once secreted, has a number of pro-inflammatory effects on activated endothelium and on phagocytes and reflects activation of the innate immune system. Calprotectin has been demonstrated to be secreted during inflammation and play a role in a number of inflammatory diseases. In view of calprotectin reflecting monocyte activation, we aimed to investigate calprotectin in patients with Familial Mediterranean Fever (FMF).

## Patients and methods

All patients were genotyped with detailed well characterised mutations. Serial levels of serum of calprotectin were measured by ELISA. Patients and healthy controls (HC) cell surface calprotectin on monocytes and neutrophils as well as intracellular peripheral blood mononuclear cells (PBMC) calprotectin expression were measured by flow cytometry (FACS). CD14 cells were isolated and following overnight incubation with or without LPS, calprotectin was measured in the supernatants by ELISA. Patient and HC CD14 cells apoptosis was compared.

## Results

Patients with FMF have greatly elevated levels of calprotectin. Median levels (range) in homozygous patients (n=87) 9039ng/ml (500-33544ng/ml), heterozygous with symptoms (n=81) median 9062ng/ml (1744-38119ng/ml), heterozygous without symptoms (n=56) 9736ng/ml (5205-19205), homozygous/compound without FMF (n=16) 34033ng/ml (range 2547-40000ng/ml). All groups were significantly greater than HCs (n=15)(p<0.001). There was no difference between the different mutations. Patients on anakinra (n=4) had persistently high levels despite controlled disease. There was a correlation between cell surface expression of calprotectin on monocytes and neutrophils (n=22) with CRP (r=0.62). Following stimulation of CD14 cells overnight with LPS, there was significantly more calprotectin detected in the supernatants in patients (n=7) than healthy control CD14 cells (n=3)(patients median 157.9ng/ml [range 90.8-313.4], HC median 90.62ng/ml [range 76.0-122.3])(p<0.05 Mann Whitney U-test). There was a trend to an increased mean florescent intensity (MFI) in intracellular monocyte calprotectin following PBMC isolation (patients n=8, HC n=4), but this didn't reach significance.

## Conclusion

Patients with homozygous mutations both with and without disease have elevated serum calprotectin which doesn't correspond to disease activity unlike some autoimmune diseases. Cell surface expression on monocytes and neutrophils is low and correlates with the CRP response. The results suggest a trend to increased intracellular calprotectin expression, and more secretion by CD14 cells following stimulation with LPS. Lastly, apoptosis of monocytes is similar between patients and HC, suggesting that increased apoptosis does not have a role in the high serum levels observed. The exact mechanism by which these patients, especially those with mutations but no clinical disease, demonstrate elevated serum calprotectin remains to be elucidated.

